# Supplementary material for: Association of Funisitis with Short-Term Outcomes of Prematurity: A Frequentist and Bayesian Meta-Analysis
Source: Antioxidants (Basel). 2023 Feb 20;12(2):534. doi: 10.3390/antiox12020534 (PMC9951960; doi:10.3390/antiox12020534)
Supplement: Supplementary file 1 [file antioxidants-12-00534-s001.zip › antioxidants-2116644-supplementary.pdf]

Supplementary Online Content

Association of funisitis with short-term outcomes of prematurity: A frequentist and Bayesian meta-analysis.

Tamara M Hundscheid, Maurice J Huizing, Eduardo Villamor-Martinez, František Bartoš, Eduardo Villamor.

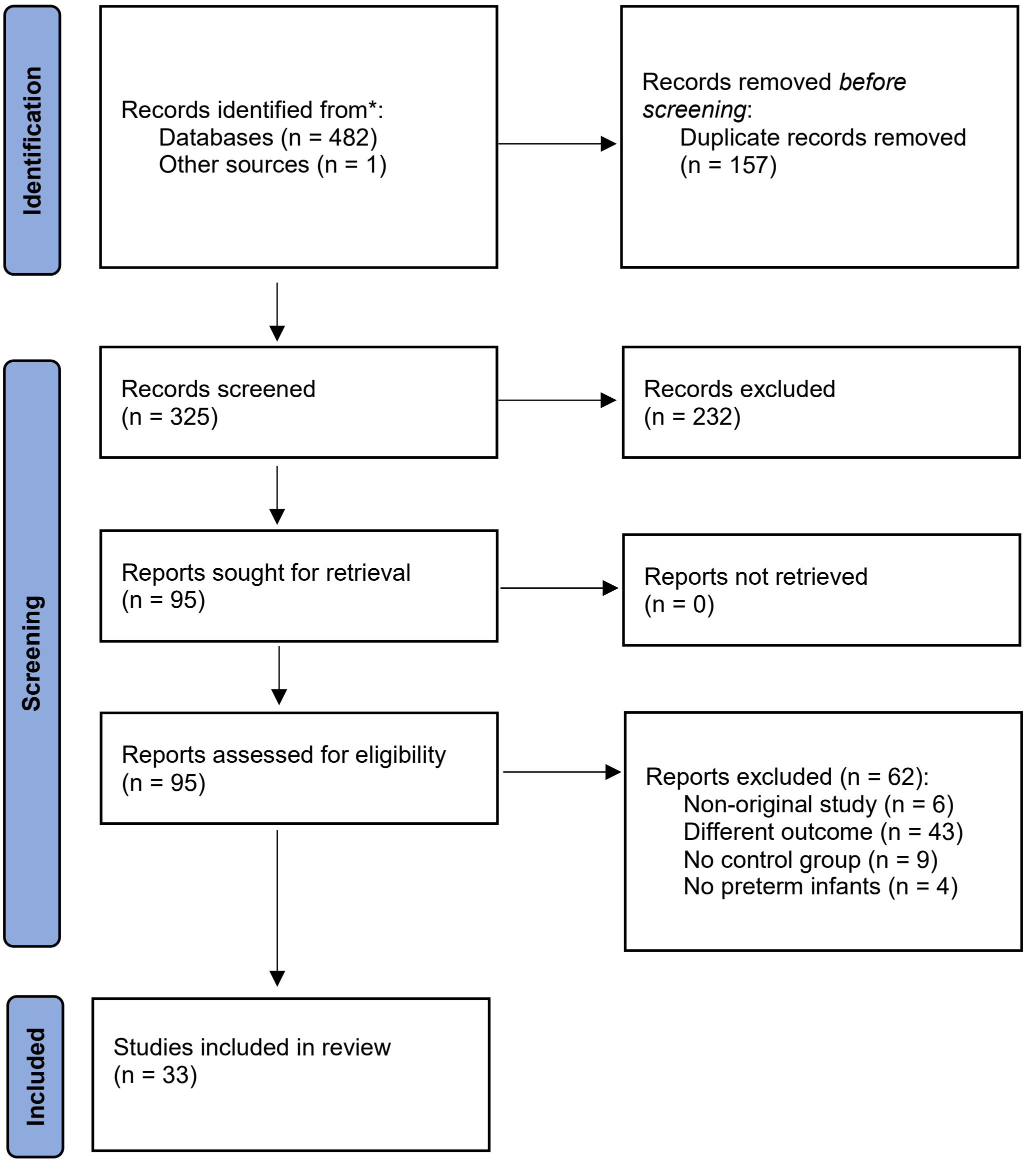

Figure S1. PRISMA Diagram

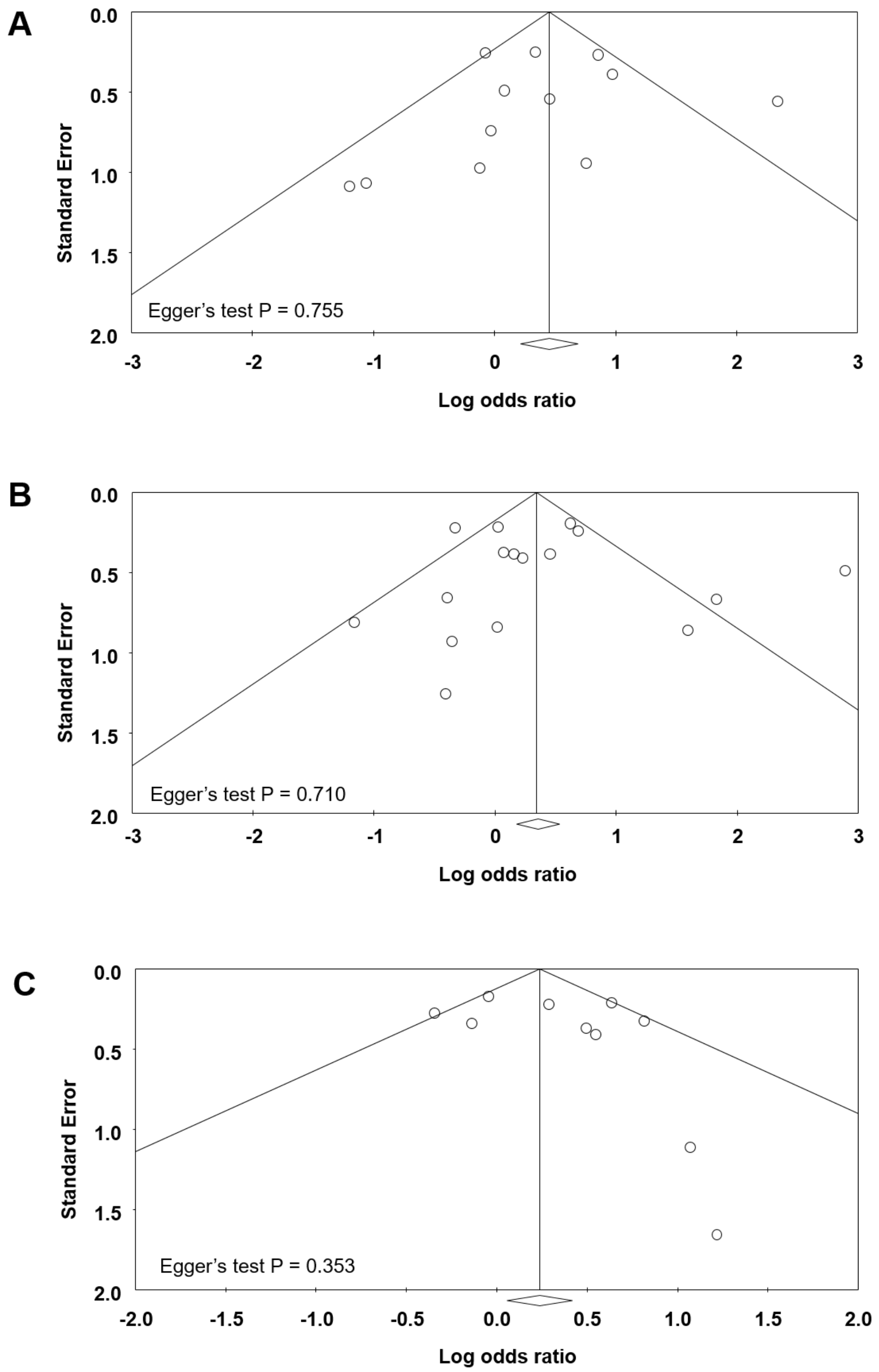

Figure S2. Publication bias: Funnel plots of exposure to funisitis compared to preterm infants without exposure to funisitis and short-term neonatal complications.

A. Funnel plot for the association with mortality (K=12)

B. Funnel plot for the association with moderate/severe bronchopulmonary dysplasia (K=16)

C. Funnel plot for the association with any sepsis (K=10)

Table S1. Characteristics of the included studies and risk of bias assessment.

First author, year	Country	Design	Prospective?	Total infants	Centers	Mean GA (weeks)	Outcomes	Selection	Comparability	Exposure/ Outcome	Total NOS
Andersen, 2003 [1]	Canada	Cohort	No	22	1	27.65	BPD	4	0	3	7
Babnik, 2006 [2]	Slovenia	Cohort	Yes	142	1	27.40	IVH	4	0	3	7
Been, 2009 [3]	Netherlands	Cohort	Yes	301	1	29.08	Mortality, BPD, PDA, PVL, NEC, IVH, sepsis	4	1	3	8
Dessardo, 2012 [4]	Croatia	Cohort	Yes	189	1	29.96	BPD, PDA, sepsis	4	0	3	7
Fujimura, 1989 [5]	Japan	Cohort	Yes	753	1	30.77	BPD	4	0	3	7
Graham, 2004 [6]	USA	Ca-co	No	300	1	27.45	PVL	4	0	3	7
Hong, 2021 [7]	South Korea	Cohort	No	474	1	32.25	Mortality, BPD, NEC, IVH, sepsis	4	2	3	9
Ikeda, 2015 [8]	Japan	Cohort	No	294	1	27.21	BPD	4	1	3	8
Jackson, 2017 [9]	USA	Cohort	No	35	3	26.48	BPD	4	0	3	7
Kelly, 2022 [10]	USA	Ca-co	No	152	NA	NA	BPD	4	0	2	6
Kent, 2004 [11]	Australia	Cohort	Yes	241	1	27.69	BPD	4	0	3	7
Kent, 2005 [12]	Australia	Cohort	Yes	212	1	27.69	Mortality, IVH	4	0	3	7
Lahra, 2009 [13]	Australia	Cohort	Yes	761	1	27.40	PDA, sepsis	4	1	3	8
Lau, 2005 [14]	Canada	Cohort	No	1296	2	33.23	Mortality, BPD, NEC, IVH, ROP, PDA, sepsis	4	1	3	8
Lee, 2017 [15]	South Korea	Cohort	No	354	1	29.00	NEC	4	2	3	9
Lee, 2015 [16]	South Korea	Cohort	No	339	1	30.04	Mortality, BPD, IVH, PVL, ROP, NEC	4	1	3	8
Liu, 2014 [17]	China	Cohort	Yes	216	1	31.69	BPD, PDA, NEC, ROP, Mortality	4	1	3	8
Lynch, 2018 [18]	USA	Cohort	No	1217	1	29.00	ROP	4	2	3	9
Pavcnik-Arnol, 2014 [19]	Slovenia	Cohort	Yes	40	1	28.73	Mortality, BPD, IVH, PVL, sepsis	4	0	3	7
Perniciaro, 2020 [20]	Italy	Cohort	No	162	1	28.81	BPD, PDA	4	0	3	7
Plakkal, 2013 [21]	Canada	Cohort	No	529	1	26.04	Mortality, BPD, PDA, NEC, sepsis	4	2	3	9
Puri, 2016 [22]	USA	Cohort	No	106	2	27.13	Mortality, NEC, sepsis	4	1	3	8

Richardson, 2006 [23]	Canada	Cohort	No	660	1	30.09	Mortality, BPD, IVH, PVL	4	0	2	6
Rocha, 2007 [24]	Portugal	Cohort	No	452	3	29.36	IVH, PVL	4	2	3	9
Salas, 2013 [25]	USA	Cohort	No	347	1	25.68	Mortality, IVH, PVL, sepsis	4	0	3	7
Smit, 2015 [26]	Netherlands	Cohort	Yes	300	2	29.37	BPD, PDA, PVL, NEC, IVH, sepsis	4	1	3	8
Thomas, 2010 [27]	Poland	Cohort	Yes	42	1	27.35	Mortality, BPD	4	1	3	8
Thorell, 2020 [28]	Sweden	Cohort	No	50	1	29.86	BPD, PDA, PVL, NEC, IVH, sepsis	4	2	3	9
Torchin, 2017 [29]	France	Cohort	Yes	1683	25	NA	Mortality, BPD	4	2	3	9
Trevisanuto, 2013 [30]	Italy	Ca-co	Yes	142	1	27.78	Mortality, BPD, NEC, IVH, PVL, sepsis	4	1	3	8
Wharton, 2004 [31]	USA	Ca-co	No	68	1	27.25	PVL	4	1	3	8
Woo, 2012 [32]	South Korea	Cohort	No	246	1	29.14	ROP	4	2	3	9
Yamada, 2015 [33]	Japan	Cohort	No	112	1	NA	Mortality, BPD, IVH, NEC	4	0	3	7

BPD: bronchopulmonary dysplasia, GA: gestational age, IVH: intraventricular hemorrhage, NA: not applicable, NEC: necrotizing enterocolitis, PDA: patent ductus arteriosus, PVL: periventricular leukomalacia, ROP: retinopathy of prematurity, USA: United States of America.

Table S2. Meta-analysis on association between funisitis and short-term outcomes of prematurity in infants with gestational age up to 32 weeks.

Outcome		K	OR	95% CI		P	Heterogeneity	
				Lower limit	Upper limit		I^2 (%)	P
Mortality		8	1.307	0.948	1.803	0.102	9.1	0.360
BPD	Any BPD	4	1.669	1.138	2.449	0.009	0.0	0.688
	Moderate/severe BPD	13	1.466	0.921	2.335	0.107	76.0	<0.001
	Severe BPD	3	1.590	0.571	4.428	0.375	81.3	0.005
	BPD or death	3	1.348	1.035	1.756	0.027	0.0	0.403
ROP	Any ROP	3	1.591	1.047	2.418	0.030	39.6	0.191
	Severe ROP	2	1.107	0.277	4.435	0.885	69.1	0.072
PDA	Any PDA	2	2.458	0.440	13.715	0.305	90.7	0.001
	PDA requiring any treatment	3	1.316	0.567	3.058	0.523	79.2	0.008
	PDA requiring surgical treatment	3	2.963	0.695	12.630	0.142	76.7	0.014
PVL	Any PVL	4	2.589	1.281	5.233	0.008	0.0	0.643
	Cystic PVL	2	0.948	0.363	2.475	0.914	0.0	0.983
IVH	Any IVH	3	2.350	1.390	3.972	0.001	31.8	0.231
	Severe IVH	6	1.585	0.926	2.711	0.093	19.4	0.287
NEC	Any NEC	1	3.424	0.660	17.770	0.143	0.0	1.000
	Severe NEC	6	1.014	0.477	2.155	0.972	57.3	0.038
	NEC or death	2	0.610	0.255	1.460	0.267	24.0	0.251
Sepsis	Any sepsis	6	1.080	0.838	1.391	0.552	24.5	0.251
	Early-onset sepsis	4	1.802	0.756	4.296	0.184	54.5	0.086
	Late-onset sepsis	4	1.089	0.775	1.532	0.623	0.0	0.396

BPD: bronchopulmonary dysplasia, IVH: intraventricular hemorrhage, NEC: necrotizing enterocolitis, PDA: patent ductus arteriosus, PVL: periventricular leukomalacia, ROP: retinopathy of prematurity.

Table S3. Data on heterogeneity of the Bayesian model-average (BMA) meta-analysis of the association between funisitis and outcomes of prematurity.

Outcome	Comparison	K	Heterogeneity (Tau)	Standard Error	Credible interval Lower Limit	Credible interval Upper Limit	BF_{rf}	Evidence for Random effects	Evidence for Fixed effects	Frequentist p-value for heterogeneity
Mortality	Fun+ vs Fun-	12	0.524	0.224	0.191	1.044	10.65	strong		0.007
	Fun+ vs Fun-CA-	12	0.546	0.255	0.183	1.158	6.169	moderate		0.004
	Fun+ vs Fun-CA+	12	0.468	0.234	0.147	1.040	0.972		weak	0.072
Any BPD	Fun+ vs Fun-	6	0.320	0.167	0.116	0.735	0.569		weak	0.683
	Fun+ vs Fun-CA-	7	0.358	0.184	0.128	0.821	0.706		weak	0.303
	Fun+ vs Fun-CA+	7	0.348	0.178	0.125	0.792	0.458		weak	0.627
Moderate/ severe BPD	Fun+ vs Fun-	16	0.723	0.217	0.368	1.204	11651	strong		<0.001
	Fun+ vs Fun-CA-	14	0.759	0.230	0.392	1.294	42766	strong		<0.001
	Fun+ vs Fun-CA+	14	0.531	0.253	0.158	1.112	2.078	weak		0.006
Severe BPD	Fun+ vs Fun-	3	0.677	0.414	0.204	1.736	4.333	moderate		0.005
	Fun+ vs Fun-CA-	3	0.789	0.455	0.242	1.952	8.420	moderate		0.002
	Fun+ vs Fun-CA+	3	0.447	0.299	0.131	1.267	0.530		weak	0.497
BPD or death	Fun+ vs Fun-	3	0.445	0.309	0.130	1.274	1.293	weak		0.403
	Fun+ vs Fun-CA-	3	0.468	0.298	0.136	1.296	1.847	weak		0.275
	Fun+ vs Fun-CA+	3	0.425	0.299	0.126	1.211	0.307		moderate	0.949
Any ROP	Fun+ vs Fun-	3	0.440	0.275	0.134	1.170	1.660	weak		0.191
	Fun+ vs Fun-CA-	4	0.384	0.231	0.129	0.978	0.938		weak	0.343
	Fun+ vs Fun-CA+	3	0.415	0.270	0.126	1.157	0.644		weak	0.541
Severe ROP	Fun+ vs Fun-	3	0.563	0.392	0.141	1.603	1.674	weak		0.106
	Fun+ vs Fun-CA-	3	0.638	0.451	0.147	1.822	2.479	weak		0.049
	Fun+ vs Fun-CA+	3	0.452	0.318	0.129	1.252	0.544		weak	0.597
Any PDA	Fun+ vs Fun-	3	0.844	0.518	0.227	2.202	15.09	strong		0.003
	Fun+ vs Fun-CA-	3	0.780	0.497	0.202	2.047	8.112	moderate		0.007
	Fun+ vs Fun-CA+	3	0.770	0.526	0.188	2.019	2.668	weak		0.014
PDA req treatment	Fun+ vs Fun-	5	0.650	0.316	0.268	1.476	4551	strong		<0.001
	Fun+ vs Fun-CA-	4	0.665	0.309	0.282	1.479	9344	strong		<0.001
	Fun+ vs Fun-CA+	4	0.429	0.247	0.142	1.051	0.784		weak	0.148
PDA req surgical treatment	Fun+ vs Fun-	3	0.843	0.604	0.172	2.447	3.299	moderate		0.014
	Fun+ vs Fun-CA-	3	0.761	0.590	0.167	2.272	1.986	weak		0.041
	Fun+ vs Fun-CA+	3	0.651	0.556	0.141	2.124	0.862		weak	0.091
Any PVL	Fun+ vs Fun-	5	0.464	0.309	0.129	1.278	0.727		weak	0.400
	Fun+ vs Fun-CA-	4	0.485	0.325	0.134	1.353	0.662		weak	0.165
	Fun+ vs Fun-CA+	3	0.514	0.399	0.133	1.518	0.708		weak	0.998

Outcome	Comparison	K	Heterogeneity (Tau)	Standard Error	Credible interval		BF_{rf}	Evidence for		Frequentist p-value for heterogeneity
					Lower Limit	Upper Limit		Random effects	Fixed effects	
Cystic PVL	Fun+ vs Fun-	5	0.351	0.193	0.119	0.848	0.303		moderate	0.996
	Fun+ vs Fun-CA-	4	0.350	0.189	0.120	0.836	0.325		moderate	0.745
	Fun+ vs Fun-CA+	3	0.471	0.315	0.134	1.334	0.591		weak	0.382
Any IVH	Fun+ vs Fun-	6	0.352	0.186	0.122	0.813	0.493		weak	0.487
	Fun+ vs Fun-CA-	6	0.341	0.174	0.121	0.779	0.419		weak	0.627
	Fun+ vs Fun-CA+	7	0.377	0.197	0.129	0.875	0.662		weak	0.451
Severe IVH	Fun+ vs Fun-	9	0.367	0.195	0.124	0.853	0.632		weak	0.425
	Fun+ vs Fun-CA-	9	0.382	0.196	0.127	0.867	0.758		weak	0.285
	Fun+ vs Fun-CA+	9	0.358	0.191	0.121	0.841	0.362		weak	0.695
Any NEC	Fun+ vs Fun-	2	0.654	0.552	0.143	2.160	0.929		weak	0.174
	Fun+ vs Fun-CA-	2	0.658	0.544	0.142	2.203	0.946		weak	0.155
	Fun+ vs Fun-CA+	3	0.533	0.402	0.131	1.616	0.728		weak	0.699
NEC stage 2 or up	Fun+ vs Fun-	8	0.623	0.276	0.231	1.283	10.85	strong		0.009
	Fun+ vs Fun-CA-	9	0.782	0.309	0.307	1.505	33.68	strong		0.001
	Fun+ vs Fun-CA+	8	0.372	0.192	0.126	0.854	0.533		weak	0.704
NEC or death	Fun+ vs Fun-	2	0.555	0.437	0.139	1.727	0.996		weak	0.251
	Fun+ vs Fun-CA-	2	0.629	0.504	0.150	1.946	1.193	weak		0.124
	Fun+ vs Fun-CA+	2	0.527	0.412	0.133	1.648	0.897		weak	0.617
Any sepsis	Fun+ vs Fun-	10	0.349	0.139	0.150	0.682	3.790	moderate		0.053
	Fun+ vs Fun-CA-	10	0.331	0.130	0.139	0.630	2.773	weak		0.077
	Fun+ vs Fun-CA+	10	0.514	0.234	0.175	1.061	3.090	moderate		0.010
EOS	Fun+ vs Fun-	6	0.559	0.290	0.177	1.271	4.281	moderate		0.017
	Fun+ vs Fun-CA-	4	0.533	0.314	0.158	1.331	2.249	weak		0.053
	Fun+ vs Fun-CA+	4	0.572	0.375	0.157	1.541	1.102	weak		0.081
LOS	Fun+ vs Fun-	6	0.330	0.165	0.120	0.760	0.466		weak	0.431
	Fun+ vs Fun-CA-	5	0.411	0.236	0.132	1.052	0.837		weak	0.195
	Fun+ vs Fun-CA+	5	0.386	0.253	0.125	0.944	0.401		weak	0.591

BF_{rf}: Bayes factor random/fixed (ratio of the probability of the data under the random effects model over the probability of the data under the fixed effect model), BPD: bronchopulmonary dysplasia, CA: chorioamnionitis, EOS: early-onset sepsis, FUN: funisitis, IVH: intraventricular hemorrhage, LOS: late-onset sepsis, NEC: necrotizing enterocolitis, PDA: patent ductus arteriosus, PVL: periventricular leukomalacia, ROP: retinopathy of prematurity.

Table S4. Meta-analysis on association between stages of funisitis and short-term outcomes of prematurity.

Outcome	Comparison	K	OR	95% CI		P-value	BMA analysis BF10
				Lower limit	Upper Limit		
Mortality	Stage 3 vs Stage 1+2	3	1.471	0.692	3.128	0.316	0.565
	Stage 2+3 vs Stage 1	2	0.646	0.243	1.719	0.382	0.611
	Stage 3 vs Stage 1	2	1.236	0.293	5.214	0.773	0.532
Any BPD	Stage 3 vs Stage 1+2	2	1.660	0.789	3.491	0.182	0.739
	Stage 2+3 vs Stage 1	2	1.637	0.924	2.903	0.091	0.935
	Stage 3 vs Stage 1	2	1.947	0.837	4.529	0.122	0.817
Severe IVH	Stage 3 vs Stage 1+2	3	0.760	0.318	1.817	0.538	0.499
	Stage 2+3 vs Stage 1	2	0.698	0.204	2.397	0.568	0.514
	Stage 3 vs Stage 1	2	0.724	0.188	2.783	0.638	0.533

BF_{10}: ratio of the probability of the data under the alternative hypothesis (H_1) over the probability of the data under the null hypothesis (H_0), BMA: Bayesian model-average, BPD: bronchopulmonary dysplasia, IVH: intraventricular hemorrhage.

Table S5. Meta-analysis on association between funisitis and levels of interleukin-6 in umbilical cord blood.

Comparison	K	Hedges' g	95% CI		P-value	BMA analysis BF10
			Lower limit	Upper limit		
Fun+ vs Fun-	3	1.065	0.505	1.625	<0.001	29.29
Fun+ vs Fun-CA-	2	0.982	0.540	1.423	<0.001	15.20
Fun+ vs Fun-CA+	2	0.688	0.164	1.212	0.010	2.896

BMA: Bayesian model-average, BF_{10}: ratio of the probability of the data under the alternative hypothesis (H_1) over the probability of the data under the null hypothesis (H_0), CA: chorioamnionitis, Fun: funisitis, NA: not applicable.

Table S6. Meta-regression of the correlation between different covariates and the odds ratio of the association of funisitis with outcome of prematurity.

Covariate	Outcome	K	Coefficient	95% CI		P	R^2-analog
				Lower limit	Upper limit		
MD in GA (funisitis minus control)	Mortality	12	0.034	-0.157	0.225	0.727	0.0
	Moderate/severe BPD	13	-0.082	-0.340	0.176	0.533	0.0
	Any sepsis	8	-0.050	-0.280	0.179	0.670	0.0
Male sex (log OR)	Mortality	11	-0.159	-1.396	1.078	0.801	0.0
	Moderate/severe BPD	11	-0.597	-1.400	0.206	0.145	0.22
	Any sepsis	9	-0.530	-1.748	0.689	0.394	0.00
Antenatal corticosteroids (log OR)	Mortality	10	0.640	-0.647	1.928	0.330	0.0
	Moderate/severe BPD	11	0.133	-0.792	1.057	0.779	0.0
	Any sepsis	9	0.380	-0.357	1.116	0.312	0.22

BPD: bronchopulmonary dysplasia, K: number of studies; R^2-analog: total between-study variance explained by the moderator.

References

1. Andersen, C.; Kent, A.; Schmidt, B.; Nahmias, C.; deSa, D.; Bourgeois, J.; et al. Pulmonary fluorodeoxyglucose uptake in infants of very low birth weight with and without intrauterine inflammation. *J. Pediatr.* **2003**, 143, 470-476; DOI: 10.1067/s0022-3476(03)00408-6.
2. Babnik, J.; Stucin-Gantar, I.; Kornhauser-Cerar, L.; Sinkovec, J.; Wraber, B.; Derganc, M. Intrauterine inflammation and the onset of peri-intraventricular hemorrhage in premature infants. *Biol. Neonate.* **2006**, 90, 113-121; DOI: 10.1159/000092070.
3. Been, J.V.; Rours, I.G.; Kornelisse, R.F.; De Krijger, R.R.; Kramer, B.W.; Zimmermann, L.J. Chorioamnionitis alters the response to surfactant in preterm infants. *Neonatology*. **2009**, 95, 375-376; DOI: 10.1016/j.jpeds.2009.07.044.
4. Dessardo, N.S.; Mustac, E.; Dessardo, S.; Banac, S.; Peter, B.; Finderle, A.; et al. Chorioamnionitis and chronic lung disease of prematurity: A path analysis of causality. *Am. J. Perinatol.* **2012, 2**9, 133-140; DOI: 10.1055/s-0031-1295654.
5. Fujimura, M.; Takeuchi, T.; Kitajima, H.; Nakayama, M. Chorioamnionitis and serum IgM in Wilson-Mikity syndrome. *Arch. Dis. Child.* **1989,** 64, 1379-1383; DOI: 10.1136/adc.64.10_Spec_No.1379.
6. Graham, E.M.; Holcroft, C.J.; Rai, K.K.; Donohue, P.K.; Allen, M.C. Neonatal cerebral white matter injury in preterm infants is associated with culture positive infections and only rarely with metabolic acidosis. *Am. J. Obstet. Gynecol.* **2004**, 191, 1305-10; DOI: 10.1016/j.ajog.2004.06.058.
7. Hong, S.; Jeong, M.; Oh, S.; Oh, J.W.; Park, C.W.; Park, J.S.; et al. Funisitis as a Risk Factor for Adverse Neonatal Outcomes in Twin Neonates with Spontaneous Preterm Birth: A Retrospective Cohort Study. *Yonsei Med. J.* **2021**, 62, 822-828; DOI: 10.3349/ymj.2021.62.9.822.
8. Ikeda, S.; Kihira, K.; Yokoi, A.; Tamakoshi, K.; Miyazaki, K.; Furuhashi, M. The levels of the neutrophil elastase in the amniotic fluid of pregnant women whose infants develop bronchopulmonary dysplasia. *J. Matern. Fetal Neonatal Med.* **2015**, 28, 479-483; DOI: 10.3109/14767058.2014.921674.
9. Jackson, C.M.; Wells, C.B.; Tabangin, M.E.; Meinzen-Derr, J.; Jobe, A.H.; Chougnet, C.A. Pro-inflammatory immune responses in leukocytes of premature infants exposed to maternal chorioamnionitis or funisitis. *Pediatr. Res.* **2017**, 81, 384-390; DOI: 10.1038/pr.2016.232.
10. Kelly, M.; Vignes, K.; Cockerham, C.; Su, L.; Stromberg, A.J.; Huang, H.; et al. Cord blood CRP: preferred biomarker to histologic chorioamnionitis for neonatal outcomes in early preterm infants. *Am. J. Obstet. Gynecol.* **2022**, 226, S94; DOI:10.1016/j.ajog.2021.11.172.
11. Kent, A.; Dahlstrom, J.E. Chorioamnionitis/funisitis and the development of bronchopulmonary dysplasia. *J. Paediatr. Child Health*. **2004**, 40, 356-359; DOI: 10.1111/j.1440-1754.2004.00366.x.
12. Kent, A.; Lomas, F.; Hurrion, E.; Dahlstrom, J.E. Antenatal steroids may reduce adverse neurological outcome following chorioamnionitis: neurodevelopmental outcome and chorioamnionitis in premature infants. *J. Paediatr. Child Health.* **2005**, 41, 186-190; DOI: 10.1111/j.1440-1754.2005.00585.x.

13. Lahra, M.M.; Beeby, P.J.; Jeffery, H.E. Intrauterine inflammation, neonatal sepsis, and chronic lung disease: a 13-year hospital cohort study. *Pediatrics.* **2009**, 123, 1314-1319; DOI: 10.1542/peds.2008-0656.
4. Lau, J.; Magee, F.; Qiu, Z.; Houbé, J.; Von Dadelszen, P.; Lee, S.K. Chorioamnionitis with a fetal inflammatory response is associated with higher neonatal Mortality, morbidity, and resource use than chorioamnionitis displaying a maternal inflammatory response only. *Am. J. Obstet. Gynecol.* **2005**, 193, 708-713; DOI: 10.1016/j.ajog.2005.01.017.
15. Lee, J.Y.; Park, K.H.; Kim, A.; Yang, H.R.; Jung, E.Y.; Cho, S.H. Maternal and Placental Risk Factors for Developing Necrotizing Enterocolitis in Very Preterm Infants. *Pediatr. Neonatol*. **2017**,58, 57-62; DOI: 10.1016/j.pedneo.2016.01.005.
16. Lee, Y.; Kim, H.J.; Choi, S.J.; Oh, S.Y.; Kim, J.S.; Roh, C.R.; et al. Is there a stepwise increase in neonatal morbidities according to histological stage (or grade) of acute chorioamnionitis and funisitis?: effect of gestational age at delivery. *J. Perinat. Med.* **2015**, 43, 259-267; DOI: 10.1515/jpm-2014-0035.
17. Liu, Z.; Tang, Z.; Li, J.; Yang, Y. Effects of placental inflammation on neonatal outcome in preterm infants. *Pediatr. Neonatol.* **2014**, 55, 35-40; DOI: 10.1016/j.pedneo.2013.05.007.
18. Lynch, A.M.; Berning, A.A.; Thevarajah, T.S.; Wagner, B.D.; Post, M.D.; McCourt, E.A.; et al. The role of the maternal and fetal inflammatory response in retinopathy of prematurity. *Am. J. Reprod. Immunol.* **2018**, 80, e12986; DOI: 10.1111/aji.12986.
9. Pavcnik-Arnol, M.; Lucovnik, M.; Kornhauser-Cerar, L.; Premru-Srsen, T.; Hojker, S.; Derganc, M. Lipopolysaccharide-binding protein as marker of fetal inflammatory response syndrome after preterm premature rupture of membranes. *Neonatology.* **2014**, 105, 121-127; DOI: 10.1159/000356735.
20. Perniciaro, S.; Casarin, J.; Nosetti, L.; Binda, C.; Salvatore, S.; Ghezzi, F.; et al. Early- and Late-Respiratory Outcome in Very Low Birth Weight with or without Intrauterine Inflammation. *Am. J. Perinatol.* **2020**, 37, S76-S83; DOI: 10.1055/s-0040-1714257.
21. Plakkal, N.; Soraisham, A.S.; Trevenen, C.; Freiheit, E.A.; Sauve, R. Histological chorioamnionitis and bronchopulmonary dysplasia: a retrospective cohort study. *J. Perinatol*. **2013**, 33, 441-445; DOI: 10.1038/jp.2012.154.
22. Puri, K.; Taft, D.H.; Ambalavanan, N.; Schibler, K.R.; Morrow, A.L.; Kallapur, S.G. Association of Chorioamnionitis with Aberrant Neonatal Gut Colonization and Adverse Clinical Outcomes. *PLoS One.* **2016**, 11, e0162734; DOI: 10.1371/journal.pone.0162734.
23. Richardson, B.S.; Wakim, E.; daSilva, O.; Walton, J. Preterm histologic chorioamnionitis: impact on cord gas and pH values and neonatal outcome. *Am. J. Obstet. Gynecol.* **2006**, 195, 1357-65; DOI: 10.1016/j.ajog.2006.03.053.
24. Rocha, G.; Proenca, E.; Quintas, C.; Rodrigues, T.; Guimaries, H. Chorioamnionitis and brain damage in the preterm newborn. *J. Matern. Fetal Neonatal Med.* **2007**, 20, 745-749; DOI: 10.1080/14767050701580515.
25. Salas, A.A.; Faye-Petersen, O.M.; Sims, B.; Peralta-Carcelen, M.; Reilly, S.D.; McGwin, G.Jr.; et al. Histological characteristics of the fetal inflammatory response associated with neurodevelopmental impairment and death in extremely preterm infants. *J. Pediatr*. **2013**, 163, 652-7.e1-2; DOI: 10.1016/j.jpeds.2013.03.081.
26. Smit, A.L.; Been, J.V.; Zimmermann, L.J.; Kornelisse, R.F.; Andriessen, P.; Vanterpool, S.F.; et al. Automated auditory brainstem response in preterm newborns with histological chorioamnionitis. *J. Matern. Fetal Neonatal Med.* **2015**, 28, 1864-1869; DOI: 10.3109/14767058.2014.971747.
27. Thomas, W.; Seidenspinner, S.; Kawczyńska-Leda, N.; Wirbelauer, J.; Szymankiewicz, M,.; Speer, C.P. Soluble receptor for advanced glycation end products (sRAGE) in tracheobronchial aspirate fluid and cord blood of very low birth weight infants with chorioamnionitis and funisitis. *Early Hum. Dev.* **2010**, 86, 593-598; DOI: 10.1016/j.earlhumdev.2010.07.013.
28. Thorell, A.; Hallingstrom, M.; Hagberg, H.; Fyhr, I.M.; Tsiartas, P.; Olsson, I.; et al. Microbial invasion of the amniotic cavity is associated with impaired cognitive and motor function at school age in preterm children. *Pediatr. Res.* **2020**, 87, 924-931; DOI: 10.1038/s41390-019-0666-3.
29. Torchin, H.; Lorthe, E.; Goffinet, F.; Kayem, G.; Subtil, D.; Truffert, P.; et al. Histologic Chorioamnionitis and Bronchopulmonary Dysplasia in Preterm Infants: The Epidemiologic Study on Low Gestational Ages 2 Cohort. *J. Pediatr.* **2017**, 187, 98-104.e3; DOI: 10.1016/j.jpeds.2017.05.019.
30. Trevisanuto, D.; Peruzzetto, C.; Cavallin, F.; Vedovato, S.; Cosmi, E.; Visentin, S.; et al. Fetal placental inflammation is associated with poor neonatal growth of preterm infants: a case-control study. *J. Matern. Fetal Neonatal Med.* **2013**, 26, 1484-1490; DOI: 10.3109/14767058.2013.789849.
31. Wharton, K.N.; Pinar, H.; Stonestreet, B.S.; Tucker, R.; McLean, K.R.; Wallach, M.; et al. Severe umbilical cord inflammation-a predictor of periventricular leukomalacia in very low birth weight infants. *Early Hum. Dev.* **2004**, 77, 77-87; DOI: 10.1016/j.earlhumdev.2004.02.001.
32. Woo, S.J.; Jung, H.J.; Kim, S.N.; Choe, G.; Ahn, J.; Park, K.H. Effects of maternal and placental inflammation on retinopathy of prematurity. *Graefes Arch. Clin. Exp.* **2012**, 250, 915-923; DOI: 10.1007/s00417-011-1648-2.

33. Yamada N, Sato Y, Moriguchi-Goto S, Yamashita A, Kodama Y, Sameshima H, et al. Histological severity of fetal inflammation is useful in predicting neonatal outcome. Placenta. 2015;36(12):1490-3. https://doi.org/10.1016/j.placenta.2015.10.021
